# Clinical Features, Treatment, and Outcomes Among Chinese Children With Anti-methyl-D-aspartate Receptor (Anti-NMDAR) Encephalitis

**DOI:** 10.3389/fneur.2019.00596

**Published:** 2019-06-06

**Authors:** Min Zhang, Wenhui Li, Shuizhen Zhou, Yuanfeng Zhou, Haowei Yang, Lifei Yu, Ji Wang, Yi Wang, Linmei Zhang

**Affiliations:** ^1^Department of Neurology, Children's Hospital of Fudan University, Shanghai, China; ^2^Department of Radiology, Children's Hospital of Fudan University, Shanghai, China

**Keywords:** anti-NMDAR encephalitis, autoimmune encephalitis, anti-N-methyl-D-aspartate receptor, children, immunotherapy

## Abstract

**Objective:** Anti-N-methyl-D-aspartate receptor (anti-NMDAR) encephalitis is the most common form of autoimmune encephalitis in pediatric patients. In this study, we aimed to investigate the clinical features and long-term outcomes of pediatric patients with anti-NMDAR encephalitis in China.

**Methods:** We conducted a retrospective study of children (age range: 0–18 years) with anti-NMDAR encephalitis treated at Children's Hospital of Fudan University between July 2015 and November 2018. Demographic characteristics, clinical features, treatment, and outcomes were reviewed.

**Results:** Thirty-four patients with anti-NMDAR encephalitis were enrolled (age range: 5 months to 14 years; median age: 7 years; female: 18). The median follow- up duration was 20 months (range: 6–39 months). Eighteen (52.9%) patients initially presented with seizures and 10 (29.4%) with abnormal (psychiatric) behaviors or cognitive dysfunction. Thirty (88.2%) patients exhibited more than two symptoms during the disease course. No neoplasms were detected. Twelve (35.2%) patients had abnormal cerebrospinal fluid (CSF) findings, including leukocytosis, and increased protein concentration. Eighteen (52.9%) patients exhibited normal brain MRI findings. Electroencephalography revealed abnormal background activity in 27 (79.4%) patients, and epileptiform discharges in 16 (47.0%) patients prior to immunotherapy. All patients received first-line immunotherapy, with 30 (88.2%) and four (11.8%) patients achieving good (Modified Rankin Scale [mRS] score of 0–2) and poor outcomes (mRS score of 3–5), respectively. Initial mRS scores differed significantly between the good and poor outcome groups. Fourteen out of 18 patients (77.7%) with seizures accepted anti-epileptic drug (AED) administration, and seizure freedom was achieved in 12 out of 14 (85.7%) patients at the last follow-up. Ten of these 12 (83.3%) patients withdrew from AED treatment within 1 year.

**Conclusions:** Most patients achieved seizure freedom, so long-term use of AEDs may not be necessary for pediatric patients with anti-NMDAR encephalitis. Among our patients, 83.3% were sensitive to first-line immunotherapy and achieved good outcomes. Higher mRS scores before immunotherapy predicted poor outcomes, highlighting the need for a comprehensive assessment of patients with anti-NMDAR encephalitis.

## Introduction

Anti-N-methyl-D-aspartate receptor (anti-NMDAR) encephalitis is a recently recognized autoimmune disorder in which auto-antibodies mainly target the NR1 subunit of the NMDA receptor, leading to a series of complex neuropsychiatric symptoms (1, 2). Reports of anti-NMDAR encephalitis have become more frequent over recent years, shedding light on the clinical characteristics of the disease. Anti-NMDAR encephalitis is a form of autoimmune encephalitis. Patients typically present with psychiatric symptoms, behavioral dysfunction, seizures, speech impairment, cognitive impairment, movement disorders, decreased consciousness, autonomic instability, and central hypoventilation. The disease is observed in patients of different ages and genders and may or may not be accompanied by ovarian teratomas or other tumors. Increased clinical recognition of this disease has led to an increase in the number of patients diagnosed with anti-NMDAR encephalitis.

Some research groups have summarized the clinical features of autoimmune encephalitis, providing a practical clinical approach to early diagnosis of the disease, rather than completely relying on the detection of autoantibodies (3, 4). Moreover, a meta-analysis found that earlier treatment of anti-NMDAR encephalitis leads to better outcomes among children (5). However, the clinical symptoms of anti-NMDAR encephalitis are complex, especially in younger pediatric patients, and many clinicians cannot promptly distinguish them from those of other diseases such as viral encephalitis or psychological conditions. Therefore, this study aimed to summarize the demographic characteristics, clinical features, ancillary examination results, treatments, and outcomes of Chinese children with anti-NMDAR encephalitis.

## Materials and Methods

This retrospective study included 34 pediatric patients with anti-NMDAR encephalitis, who were diagnosed at the Department of Neurology at Children's Hospital of Fudan University (Shanghai, China) between July 2015 and November 2018. The study was approved by the Ethics Committee of the Children's Hospital of Fudan University, which waived the requirement for informed consent owing to the retrospective nature of the study.

All patients met the following inclusion criteria: (a) met the diagnostic criteria for definite anti-NMDA receptor encephalitis (3); (b) treatment with first-line immunotherapy during the acute phase, including methylprednisolone and/or immunoglobulin and/or plasma exchange; (c) age between 0 and 18 years; and (d) duration of follow-up exceeding 6 months, with complete medical records. We excluded patients with other possible etiologies such as viral encephalitis or psychological conditions.

Medical information was collected from medical records or via telephone interviews and follow-up was continued until the patient died or was lost to follow-up. We reviewed patients' clinical data, including age, gender, age at disease onset, follow-up duration, initial symptoms, duration between symptom onset and diagnosis, duration between symptom onset and immunotherapy, CSF examination results, brain magnetic resonance imaging (MRI) results, results of screenings for systemic neoplasms, electroencephalography (EEG) findings, and treatment strategies. Serum and CSF samples from each patient were sent to Oumeng Biotechnology Corporation (Shanghai, China) to screen for antibodies against the NMDA receptor. All samples were evaluated for anti-NMDAR IgG antibodies via indirect immunofluorescence using EU 90 cells transfected with the NMDAR1 subunit (NR1) of the NMDAR complex and immobilized on BIOCHIPs (Euroimmun AG, Lubek, Germany). CSF leukocytosis was defined as white cell count >5/mm^3^ while elevated CSF proteins 450 >mg/L. Tumor screening (MRI and/or CT and/or ultrasound of the chest, abdomen, and pelvic cavity) was performed once each patient was diagnosed with anti-NMDAR encephalitis. All patients were screened for tumors regularly after discharge, including MRI of the chest, abdomen, and pelvic cavity (once a year for children >12 years and biennial for children <12 years of age).

Digital-video EEG records were obtained at least once before immunotherapy, three to 6 months after immunotherapy, and at the last available follow-up. EEG data were recorded for at least 30 min. All EEG recordings were retrospectively evaluated by a pediatric epileptologist familiar with the patient's age and diagnosis, but not with his/her clinical state, symptoms, or signs. EEG data were categorized as follows: background activity (normal, generalized slowing, focal slowing, and extreme delta brushes [EDB]); interictal epileptic paroxysms such as sharp waves, spike waves, polyspike waves, or generalized discharges; focal discharges; and multifocal interictal epilepticdischarges.

Brain MRI findings were obtained from all patients before immunotherapy. Abnormal brain MRI findings were defined as hyper intensities on T2-weighted images (T2WI), fluid-attenuated inversion recovery (FLAIR) images and/or hypo intensities on T2-weighted images (T1WI). The same pediatric neurologist reviewed all the brain MRI results.

Outcomes were evaluated based on mRS scores. After discharge, outcome evaluations were performed during clinical visits to the neurologist or via telephone follow-up. The evaluation standards were as follows: full recovery, mRS score of 0; mild deficits, mRS scores of 1–2; severe deficits, mRS scores of 3–5; or death, mRS score of 6.

We used SPSS version 19.0 for all statistical analyses (SPSS Inc., Chicago, IL, USA). Continuous variables such as age, the interval from symptom onset to definitive diagnosis, and the interval from symptom onset to immunotherapy were analyzed using independent *t*-tests. Categorical variables were analyzed using Fisher's exact test, and ordinal variables were analyzed using Fisher–Freeman–Halton tests. *P* < 0.05 (two-sided) were considered significant.

## Results

### Clinical Characteristics

**Figure 1:**
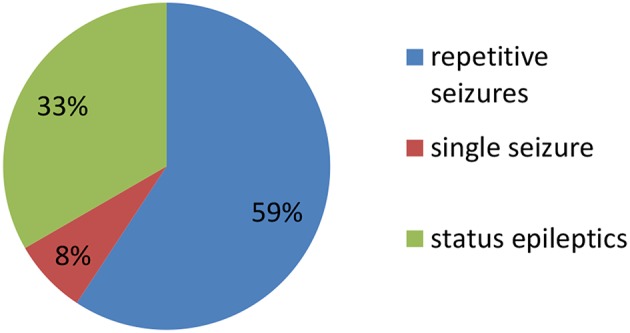
Percentage of patients with repetitive seizures, single seizure, and status epilepticus (SE).

**Figure 2:**
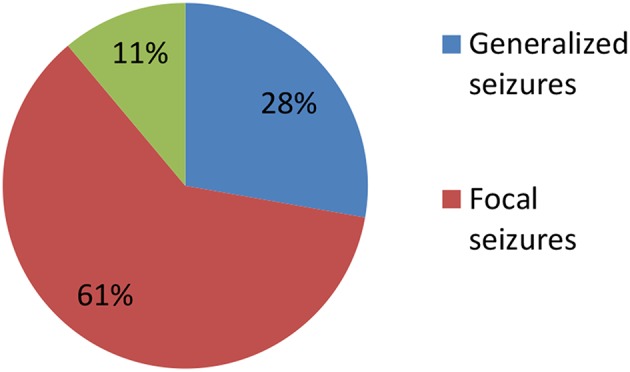
Percentage of patients with focal, generalized, and both focal and generalized seizures epilepticus (SE).

Clinical characteristics of the 34 included patients are presented in Table 1. Patients' ages ranged from 5 months to 14 years (median age: 20 months), and 18 were female (52.9%; a female-to-male ratio of 1.125). Thirty patients (88.2%) were younger than 12 years of age, and 14 patients (41.2%) were younger than 6 years of age at symptom onset. The median follow-up duration was 20 months, ranging from 6 to 39 months. The initial presentation included seizures in 18 patients (52.9%), abnormal (psychiatric) behaviors or cognitive dysfunction in 10 patients (29.4%), a movement disorder in 3 patients (8.8%), and a decreased level of consciousness in 3 patients (8.8%). Thirty patients (88.2%) developed at least two of the six symptom categories over the course of their disease. Three patients (8.8%) were hospitalized in intensive care unit (ICU) due to central hypoventilation, coma, or refractory seizures, respectively. Each of the 18 patients who experienced seizures had onset during the acute phase of anti-NMDAR encephalitis, which was defined as <3 months after symptom onset. Seizure types included repetitive seizures (16/18, 88.8%), single seizures (2/18, 11.1%), and status epileptics (9/18, 50%) (Figure 1). Generalized and focal seizures were noted in 5 (27.7%) and 11(61.1%) of 18 patients, respectively (Figure 2). Only two (11.1%) patients reported seizures at the last follow-up. No patients developed tumors or died during follow-up.

**Table 1:** Patients' demographic and clinical characteristics.

**Item**	**All patients (%)**	**Age under 12 (%)**	**Age under 6 (%)**
Number	34	30	14
Female: male	18:16	16:14	8:6
Median age, range(months)	86 (5–171)	81 (5–136)	32 (5–67)
**INITIAL SYMPTOMS**			
Psychiatric/behavior	8 (23.5%)	8 (26.7%)	4 (28.6%)
Seizure	16 (47.1%)	13 (43.3%)	10 (71.4%)
Others	10 (29.4%)	9 (30%)	0

### Ancillary Examination Results

Initial CSF findings before immunotherapy are shown in Table 2. Eleven patients (32.4%) had CSF pleocytosis, seven (20.6%) had increased protein concentrations only, and six (17.6%) had both. Anti-NMDAR antibodies were identified in CSF obtained from 9 patients (26.5%) and both serum and CSF of 25 patients (73.5%). No patients were positive for anti-NMDAR antibodies in serum only. Brain MRI findings were normal in 19 (55.9%) of 34 patients. The remaining 15 patients (44.1%) exhibited abnormalities that included increased signal on T2WI or FLAIR images (*n* = 14, two in the temporal lobes, one in the frontal cortex, three in the thalamus, one in the parietal lobe, seven in the cerebral cortex/gray matter) and encephalomalacia (*n* = 1).

**Table 2:** Results of ancillary examinations.

**Examinations**	**All patients**
Brain MRI	Numbers (%)
Total abnormal findings	18 (52.9%)
EEG	
Abnormal background	
Before immunotherapy	27(79.4%)
3–6months after immunotherapy	2(5.9%)
Last follow up	0(0%)
Interictal Epileptiform Discharge	
Before immunotherapy	16 (47.0%)
3–6months after immunotherapy	5 (14.7%)
Last follow up	2 (5.8%)
EDB	2 (5.8%)
CSF Results	
Abnormal findings	12 (35.3%)
Pleocytosis	11 (32.4%)
Increased protein concentration	7 (20.6%)
Pleocytosis and increased protein concentration	6 (17.6%)
Positive OB(Total number 11)	1 (9.1%)

*EEG, electroencephalogram; EDB, extreme delta brush; CSF, cerebrospinal fluid; OB, Oligoclonal band*.

**Figure 3:**
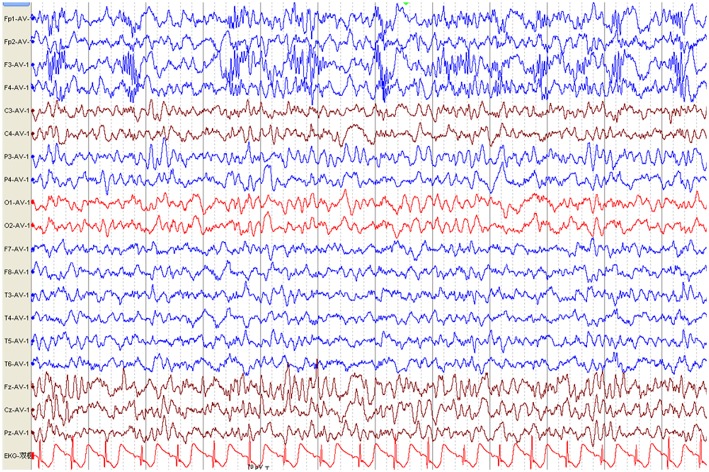
Electroencephalography pattern of a 4-year and 5-month-old female child with anti-N-methyl-D-aspartate receptor encephalitis (Case 8), who presented with clonic seizures of her right limbs as her initial symptom. EEG was recorded 43 days after symptom onset and before immunotherapy. EEG shows bilateral frontal-predominant fast activity at 20–30 Hz riding on the generalized rhythmic delta activity (EDB).

**Figure 4:**
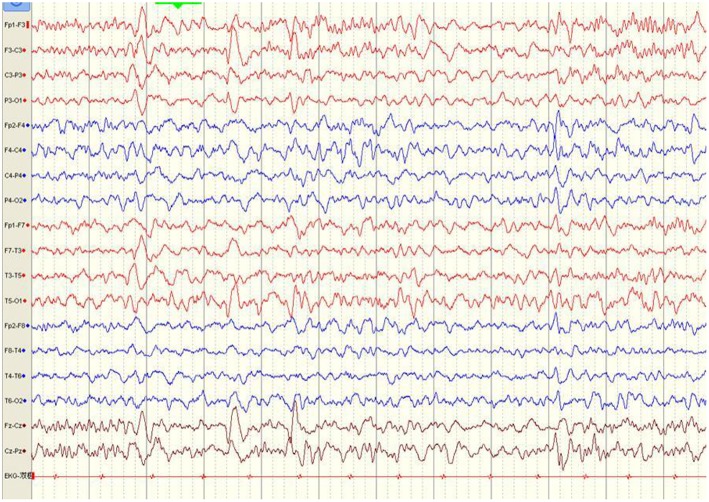
Normal EEG was recorded six months after immunotherapy (Case 8).

The first available EEG findings detected before immunotherapy included generalized slowing in 25/34 (73.5%) patients and focal slowing in 2/34 (5.9%). Normal background activity was observed in only 7/34 (20.6%) patients, and in 32/34 (94.1%) patients 3 months post-immunotherapy, and in all patients 9 months post-immunotherapy. Sixteen of 34 (47.1%) patients reported interictal epileptic paroxysms during the acute stage of anti-NMDAR encephalitis. This rate decreased to 14.7% (*n* = 5) 3–6 months after immunotherapy and 2.9% (*n* = 1) at the last follow-up. EDB patterns were recorded in 2/34 (5.9%) patients (Figure 3) and disappeared 6 months after immunotherapy (Figure 4).

### Treatments and Outcomes

**Figure 5:**
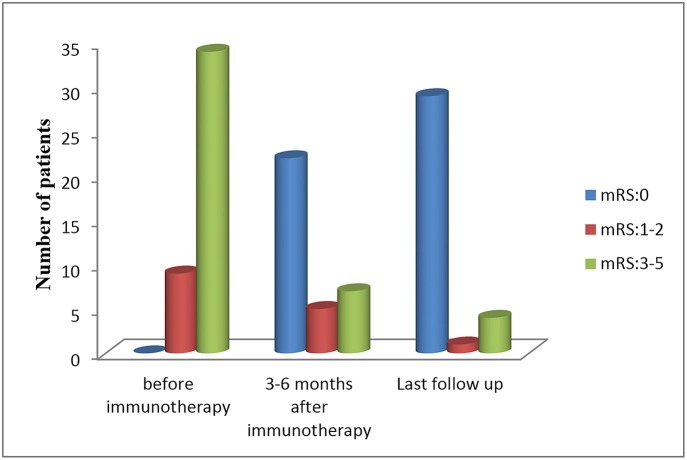
Patients' mRS scores during the follow-up period.

All patients received first-line immunotherapy, including intravenous methylprednisolone, intravenous immunoglobulin, plasma exchange, or an arbitrary combination of these treatments. The median interval between symptom onset and the start of immunotherapy treatment was 23.9 days, ranged from 7 to 42 days. The median duration of follow-up was 20 months, with a range of 6 to 39 months. Twenty-five of the 34 (73.5%) patients were treated within 30 days of first symptom appearance. Three patients (8.8%) were exclusively treated with intravenous methylprednisolone (15–30 mg/kg per day for 3–5 days), 29 patients (85.3%) with both intravenous methylprednisolone and intravenous immunoglobulin (IVIG, 0.4 g/kg per day for 5 days or 1 g/kg per day for 2 days), and two patients (5.9%) with a combination of intravenous methylprednisolone, IVIG, and plasma exchange. The median mRS score before immunotherapy was 5, which decreased to zero following 3–6 months of initial immunotherapy (Figure 5). By the last follow-up, 29 patients (85.2%) had fully recovered, one patient (2.9%) exhibited mild deficits (weakness on one side of the body), and four patients (11.8%) exhibited severe deficits (one with speech disturbances and memory deficits, one with dyskinesia, and two with intractable epilepsy). No deaths were noted at the last follow-up.

### Comparison Between the Good and Poor Outcome Groups

Table 3 shows between-group comparisons of the good and poor outcome groups. The initial median mRS score was significantly higher in the poor outcome group than in the good outcome group (*p* = 0.014). Initial symptoms, CSF findings, the median age at the appearance of initial symptoms, the median interval between onset and diagnosis, the median interval between onset and immunotherapy, MRI findings, EEG findings, and ICU admission showed no significant between-group differences.

**Table 3:** Comparison of the good and poor outcome groups.

**Item**	**Good outcome**	**Poor outcome**	***P*-value**
Patient number	30	4	/
Initial syndrome			/
Seizure	15	3	0.9467
Abnormal (psychiatric) behavior or cognitive dysfunction	9	1	0.7723
Others	6	0	0.5289
Abnormal CSF finding	12	2	0.8219
Median age(m)	99	24	0.1772
Median initial mRS	5	5	0.0141
Median interval between onset and diagnosis(d)	22	30	1.0000
Median interval between onset and immunotherapy(d)	20	29	0.6721
Abnormal MRI findings	12	3	0.3891
Abnormal EEG background	25	2	1.0000
Abnormal interictal epileptic discharges	14	2	0.5671
ICU stay	3	0	0.9290

### Comparison Between Patients Younger and Older Than 6 Years old

Table 4 shows the comparison between patients younger than 6 years old and older than 6 years. We observed no significant between-group differences for initial symptoms, CSF findings, median initial mRS score, the median interval between onset and diagnosis (d), the median interval between onset and immunotherapy (d), MRI findings, interictal epileptic discharges, or ICU admission.

**Table 4:** Comparison of patients younger than 6 years old with those older than 6 years old.

**Item**	**Age under 6 y**	**Age older than 6 y**	***P*-value**
Patient number	14	20	/
Initial syndrome			/
Seizure	10	8	0.6835
abnormal (psychiatric) behavior or cognitive dysfunction	4	6	1.0000
Others	0	6	0.0717
Abnormal CSF finding	4	8	0.7477
Median age(m)	36.5	110	0.0000
Median initial mRS	5	5	0.9830
Median interval between onset and diagnosis(d)	29.5	19	0.1010
Median interval between onset and immunotherapy(d)	27.5	17	0.0980
Abnormal MRI findings	7	8	0.8204
Abnormal EEG background	11	16	1.0000
Abnormal interictal epileptic discharges	9	7	0.1820
ICU stay	2	1	0.7450

## Discussion

Previous research has demonstrated that there are more cases of anti-NMDAR encephalitis than other kinds of autoimmune encephalitis, and that early diagnosis and aggressive medical management decrease the likelihood of morbidity and mortality (6–9). Therefore, if anti-NMDAR encephalitis is suspected and other diseases can be ruled out, treatment should begin as early as possible (10). However, there are significant differences in clinical features between pediatric and adult patients. Here we retrospectively analyzed the clinical characteristics, ancillary examination results, and outcomes of Chinese pediatric patients with anti-NMDAR encephalitis.

We observed no significant differences in sex in the present study. These findings correspond to the findings of a previous study that focused on pediatric patients with anti-NMDAR encephalitis in south-central China (11). In children, psychiatric syndromes can present as abnormal behaviors or cognitive dysfunction. This is particular true for preschool-aged children, because it is difficult for them to describe their symptoms and emotional states. Therefore, we could not make objective judgments regarding cognitive function, including the presence of memory deficits, which are independently associated with poorer outcomes (12). Instead, we attributed psychiatric symptoms and abnormal behaviors or cognitive dysfunction to a single category of symptoms. Eighteen of the 34 included patients (52.9%) initially presented with seizures, while 10 (29.4%) presented with abnormal (psychiatric) behaviors or cognitive dysfunction. We concluded that seizures and abnormal (psychiatric) behaviors and cognitive dysfunction are the most common symptoms of pediatric anti-NMDAR encephalitis, in agreement with previous findings (13–16).

Armangue et al. (9, 14) reported that younger children with anti-NMDAR encephalitis typically presented with neurologic symptoms, whereas adolescents more often presented with psychiatric symptoms. However, in our study, 75% (3/4) of adolescents presented with seizures as their initial symptom, and there was no significant difference in initial symptoms between older and younger patients (Table 4). This difference may be attributed to the relatively small sample of adolescents in our study. According to the literature, adult patients more frequently present with focal seizures, while children more frequently present with generalized seizures that develop into the dominant seizure type over the course of the disease (17). In a recent study of 17 pediatric patients with anti-NMDAR encephalitis, generalized seizures were reported in 5/16 patients (31%), while focal seizures were reported in 4/16 (25%) patients, another 7/16 (44%) patients had both generalized, and focal seizures (16). In our study, among 18 patients experienced seizures, 11 (61.1%) presented with focal seizures, five (27.7%) with generalized seizures, and two (11.1%) with both types. Age-related differences in patients' constitutions and the use of video-EEG to determine seizure type may explain these differences.

Of the 14 patients treated with AEDs, 12 (85.7%) got seizure free during the acute stage of anti-NMDAR encephalitis, and 10 patients (71.4%) were able to withdraw from AEDs within 1 year. At the final follow-up, only two patients (14.3%) with ongoing epilepsy were treated with AEDs, indicating that long-term use of AEDs may not be necessary for pediatric patients with anti-NMDAR encephalitis. Similar results have been reported in previous studies involving both adult and pediatric patients (18). No tumors were detected in our study, which demonstrates that younger age is associated with a lower rate of teratomas (2, 11).

Although not generally helpful in diagnosing anti-NMDAR encephalitis, imaging studies play a key role in the workup of patients with suspected anti-NMDAR encephalitis because these modalities can rule out other conditions that could create a similar neurologic picture (19). A recent study demonstrated that anti-NMDA encephalitis is an autoimmune-mediated disease without specific brain MRI features. The authors categorized the brain MRI findings of patients with anti-NMDA receptor encephalitis into four types. Of these, hippocampal lesions were the most common brain abnormalities and were identified as risk factors contributing to poor prognosis (20), consistent with previous reports (21, 22). However, as with our results, some research has indicated that abnormal MRI findings do not affect prognosis as indicated by mRS scores (23). In our study, 55.9% of patients had normal brain MRI findings, and none exhibited hioppocampal lesions. This discrepancy may be due to differences between pediatric and adult patients, or to the relatively small sample size of our study. Therefore, future studies with larger samples are needed to compare brain MRI features between pediatric and adult patients.

Previous studies have indicated that the parietal aEEG bandwidth may separate patients with favorable and poor long-term outcomes in the early disease stages (24). In our study, the first available EEG findings that obtained before immunotherapy showed generalized slowing in 14/18 patients (77.7%), focal slowing in 2/18 patients (11.1%), and no abnormalities in 2/18 patients (11.1%), consistent with the findings of previous reports (25, 26). No patients exhibited abnormal background activity at the final follow-up. These findings suggest that generalized slowing of EEG background activity is an important clue to diagnosing anti-NMDAR encephalitis during the acute stage, but it is not specific to anti-NMDAR encephalitis. Recent studies have demonstrated that the presence of EDB patterns is a marker of more severe disease among patients with anti-NMDAR encephalitis and corresponding with worse outcomes (27). Past researchers observed EDB patterns on EEG in 33% of patients with anti-NMDAR encephalitis (28). However, in our study, EDB patterns were detected in only 2/18 (11.1%) patients, likely due to differences in the time of EEG recording and individual differences in patients within the various study groups. Since prompt diagnosis is crucial (29), we recommend use of video-EEG monitoring for all patients with suspected anti-NMDAR encephalitis (30). Nonetheless, a recent study suggested that EDB is also not unique to anti-NMDAR encephalitis and can occur (albeit rarely) in patients with mesial temporal lobe epilepsy. While the presence of EDB should prompt suspicion of anti-NMDAR encephalitis, other possible etiologies should not be ignored (31).

Some previous studies have suggested that the prognosis is poor among patients with severe anti-NMDAR encephalitis (2, 32), but the long-term prognosis of anti-NMDAR encephalitis is good (33, 34), even in patients whose diagnoses were missed or in those with prolonged diagnostic delays who eventually recovered or substantially improved (35). Predictors of poor outcomes included younger age, decreased consciousness, memory deficits, ICU admission, treatment delay >4 weeks, lack of clinical improvement within 4 weeks, abnormal MRI, and CSF white blood cell count >20 cells/μL, etc. (2, 36–38). In our study, higher initial mRS scores predicted poor outcomes, in accordance with Anastasia Zekeridou et al. (39). However, our sample size was relatively small and further studies involving larger sample sizes are required to determine the risk factors for poor prognosis in this patient population.

According to our experience, most patients with anti-NMDAR encephalitis continue to improve within 2 years or longer, even when treated with first-line immunotherapy alone. For patients with slow clinical improvement, first-line immunotherapy can be re administered. In this study, some caretakers refused second-line immunotherapy because of cost concerns or concerns over clinical side effects, so all our patients were treated with first-line immunotherapy. Although no patients attained mRS scores of 0–2 (0%) before immunotherapy, 83.3% of them attained such scores at the final follow-up. This result indicated that first-line immunotherapy is an effective measure for pediatric patients with anti-NMDAR encephalitis. Besides, our patients' good outcomes may be associated with admission to the less intensive care unit and prompt immunotherapy after diagnosis with anti-NMDAR encephalitis.

In a recent study involving 111 patients with anti-NMDAR encephalitis, 39 (35.1%) patients were included in the severe group. Even patients with the most severe forms of anti-NMDAR encephalitis can eventually achieve good long-term outcomes after receiving early, positive, and unremitting combined immunotherapy and life support (25). Another study (40) involving 19 children with anti-NMDAR encephalitis in Thailand revealed that IVIG treatment, was associated with greater improvements in mRS scores. These findings underscore the benefits of IVIG treatment for this condition. Zhang et al. (13) analyzed the individual outcomes associated with three first-line immunotherapies and combinations of any two immunotherapies. Their findings revealed that patients treated with a combination of corticosteroids and IVIG plus second-line immunotherapy more frequently achieved full recovery than patients treated with a combination of corticosteroids and IVIG. Second-line immunotherapy with rituximab, cyclophosphamide, or both significantly improved outcomes in patients who did not respond to first-line therapy and decreased the frequency of relapses (2). Therefore, second-line immunotherapy may be necessary when patients do not achieve full recovery with first-line immunotherapy only.

Nonetheless, some recent studies have reported substantial deficits across multiple cognitive domains and behavioral problems in both adult and pediatric patients (41–44). These findings indicate that, even when good outcomes are achieved, full recovery may not be possible. Alternatively, while mRS scores are effective tools for assessing disability in patients with stroke, these scores may not be the most suitable tool for evaluating outcomes in patients with anti-NMDAR encephalitis who present with seizures and abnormal (psychiatric) behaviors or cognitive dysfunction. This is particularly true for infants who cannot walk or express their emotions. Future studies should seek to determine the most appropriate method for comprehensively assessing cognitive and social functions in patients with anti-NMDAR encephalitis at different ages.

Our study had several limitations. The functional status assessment may be susceptible to recall bias given the retrospective nature of the study. Our cohort only included patients diagnosed and treated at the Children's Hospital of Fudan University in Shanghai, which may also have introduced a selection bias. All patients were treated with first-line immunotherapy so we could not assess differences in the effects between first-line immunotherapy and other immunotherapy measures. The relationship between anti-NMDAR antibody titers and clinical symptom severity or outcomes was not examined and should be a focus of future studies.

## Conclusion

In our study, seizure freedom was typically achieved by the final follow-up, indicating that long-term use of AEDs may not be necessary for patients with anti-NMDAR encephalitis. More than half of the patients exhibited normal brain MRI findings. Our results further indicated that generalized slowing of EEG background activity is the main characteristic of pediatric anti-NMDAR encephalitis during the acute stage. In addition, 83.3% of our patients were sensitive to first-line immunotherapy and achieved good outcomes. Higher mRS scores before immunotherapy predicted poor outcomes among pediatric patients with anti-NMDAR encephalitis. Future studies should aim to determine the most appropriate methods for comprehensively assessing cognitive and social functions in patients with anti-NMDAR encephalitis, particularly infants.

## Ethics Statement

This study was approved by the Ethics Committee of Children's Hospital of Fudan University, who waived the requirement for informed consent due to the retrospective nature of the study.

## Author Contributions

MZ and WL wrote the initial draft of the paper. JW and LY acquired patients' demographic and clinical data through medical records and telephone interviews. YZ guided the analysis of EEG signals. HY guided the analysis of MRI and CT results, SZ guided the study design, and LZ and YW made critical revisions to the manuscript.

### Conflict of Interest Statement

The authors declare that the research was conducted in the absence of any commercial or financial relationships that could be construed as a potential conflict of interest.
